# Human Temperaments. VII.—The Religious Temperament


**Published:** 1916-07-22

**Authors:** 


### HUMAN TEMPERAMENTS.

### VII.—The Religious Temperament.*

THE root of religion is sacrifice: the primary purpose of sacrifice is propitiation. In primitive religions the gods are frankly malignant. They are inspired by malignity, and only by sacrifices can they be propitiated and induced to forgo the execution of their malevolent designs. As men become more enlightened, the malignity of their gods becomes mollified, and at length Christianity attempts to depict the Deity not as an avenger, for ever on the watch to inflict pain and suffering in revenge for offences committed against Him, but as a loving Father, desiring only the welfare of His votaries. This is the professed ideal of the Christian religion, but it is never maintained. At the one extreme the Roman Catholics teach the necessity of propitiation by penances interspersed among the pleasures of life; at the other extreme, the Puritans teach the necessity of propitiation by renouncing pleasures altogether.

### VICARIOUS AND SELF SACRIFICE.

The religious temperament consists, therefore, in proneness to sacrifice, which is evinced in two ways—self-sacrifice and vicarious sacrifice; and, accordingly, the religious temperament is twofold. At the one extreme we see St. Simeon Stylites and the noble army of self-martyrs, the self-torturer, the hermit, the Trappist, the monk, the nun, and so on down to the High Church lady who deprives herself of sugar in her tea during Lent, or rises at an inconvenient hour to attend early celebration. At the other extreme is the savage who immolates his human victim on the altar, the inquisitor who tortures his heretic to propitiate his Deity, and the Puritan who deprives his children of innocent pleasures for the same reason. No doubt in many cases the two forms of sacrifice are practised by the same person. No doubt Abraham in intending to sacrifice his only son was torn to the heart with anguish at the necessity; no doubt Torquemada inflicted penances on himself; and the Puritan father who deprives his children of their innocent little pleasures may deprive himself also of pleasures equally innocent; but the vicarious sacrifice is so much less unpleasant that, human nature being what it is, it is apt to become preponderant; and as sacrifice, after all, is sacrifice, it may seem superfluous, and tending to foster self-righteousness and spiritual pride, to cultivate both forms. Hence the pleasures that the Puritan denies himself are apt to be those which even if indulged in would yield him little pleasure, while those of which he deprives others inflict very real mortification. His abstention from dancing, at his age and with his portly figure, is no great sacrifice to him; but his prohibition of the same exercise for his children is for them a real deprivation.

The preponderance of vicarious sacrifice to self-sacrifice cannot take place without a certain callousness on the part of the sacrificer to the sufferings of others, and callousness is a half-way house on the road to cruelty; and thus is explained that streak of cruelty which is so frequent an accompaniment, and, indeed, a manifestation of the religious temperament. Not all persons of this temperament are cruel; far from it. Many are of the most benevolent and amiable disposition, but these are not addicted to vicarious sacrifice. They sacrifice themselves. They give freely of their money, their time, their labour, their thought, their convenience and comfort, either to forward the welfare of others or *ad majorem Dei gloriam*; but they do not demand sacrifices from others. Rather, by their own unselfishness and readiness to sacrifice themselves, they foster selfishness in those around them. It is those who make vicarious sacrifices, those who demand and exact sacrifices from other people, and especially those who make themselves the instruments of other people's sacrifices, that are apt to become first callous and then cruel, and at length may develop or degenerate into such monsters of cruelty as Torquemada, Bishop Bonner, John Calvin and John Wesley. Not satisfied with the sufferings they are able to inflict and to witness, their malignant imaginations revel in describing the torments of hell, to which they hope rather than fear that their victims will be consigned. Cruelty is not a necessary part of the religious temperament, but it is a frequent accompaniment of this temperament; and it is found, as has been said, in those who prefer to make vicariously the sacrifices that are the root element in religion.

### SELF-SACRIFICE AS A VICE.

Those examples of the religious temperament that tend to self-sacrifice provide us with some of the most beautiful and admirable specimens of human character, just as those that tend to vicarious sacrifice provide us with some of the worst; but even self-sacrifice may be pushed to excess and will then become a vice. Even those beautiful characters that compel our admiration by their unselfish devotion to others are not always beneficial to these others. The more a person sacrifices himself, or more often herself, to ministering to the comfort and welfare of others, the more likely is he or she to breed selfishness in those others; and in experience we find that the most selfish people are those upon whom unselfishness has been most lavished. Self-sacrifice does not, however, always take the form of increasing the comfort of others by renouncing our own. In many cases there is mere renunciation for its own sake, or, rather, for the sake of compensations that are expected to accrue after this life; and it is in these

* The previous articles appeared on May 6, 13, 27, June 10 and 24, and July 8, 1916; and a letter on the subject on May 20.

that we see the most extreme degrees of self-sacrifice—of self-sacrifice that often amounts to self-torture. As it is difficult to withhold our admiration from the self-sacrifice of unselfishness, even though its effect may be to increase the selfishness of others, so it is difficult to bestow admiration upon this kind of self-sacrifice, which is in many cases but a refined and excessive selfishness. The monk and the nun who renounce the pleasures and comforts of what they are pleased to call the world, and who renounce them, not in order to be of service to their fellow men and women, but in order to shirk the burden and the battle of life, and to secure for themselves a better future in the world to come, do not command our admiration or our sympathy. They are engaged in a commercial transaction which they believe will turn out to their profit, and we admire them no more than we admire the trader who embarks on a speculation for the sake of the return that it will bring; or, if we do, the admiration is rather for their astuteness than for their virtue.

### Religion and Morality.

Religion and morality are very different things, and although the man of religious temperament may be a moral man, true and just in all his dealings, there is no necessity that he should be so, nor is it at all necessary that a highly moral and virtuous man should be religious. Religion and morality have no more necessary connection than blueness and hardness. The sea is blue, but it is not hard, and the diamond is hard, but it is very rarely blue. No one can doubt that the Duke of Alva, Louis XI., Louis XIV., Charles V., and James II. were extremely religious, and no one can doubt that every one of these historic characters was a scoundrel. On the other hand, Julian the Apostate, though by no means a religious man, was far from the worst of the Roman Emperors, and many a philosopher whose life was blameless, and who is revered as a model of virtue, has had no religion at all.

Neither is intelligence any monopoly of the religious temperament: there are as many stupid people as clever people who are religious, and as many clever people as stupid people who are not. There is, indeed, a certain antagonism between reason and the religious temperament. Those who possess this temperament in high degree are apt to flout the processes and results of reasoning, and refuse to admit their validity in the realm of religion, and many very able men of religious temperament refuse to employ their intellect on religious problems.

The religious temperament is, however, strongly addicted to forms, and strongly infused with emotion. Devotion to form grows with the age of the religion, and the oldest religions not only are the most formal, but also come at length to consist mainly, and at last entirely, of forms. Each religious revolt is a revolt against the rigidity of form, but no sooner is form abolished than it settles down again and continually grows stronger. Ritual, liturgy, observance, convention are characteristic of all religions, and the older the religion the more it is bound in these forms. Even the Quakers, who professed to throw off all forms, substituted new forms for those they abolished. They abolished all peculiar ecclesiastical costume, only to institute a conventional costume of their own; they abolished the liturgical mode of addressing the Deity, only to introduce a conventional mode of addressing their fellow-men. If they have now abandoned both their conventional costume and their conventional mode of address, it is not because their religion is become less formal, but because they have become less religious.

The religious temperament is an emotional temperament. It is diffused and surcharged with emotion, which finds ready and enthusiastic expression. This does not mean that every professional religious person, every priest, parson, minister, monk, nun, dervish, fakir, and bonze is emotional, for many persons who adopt religion as a profession are not of the religious temperament, just as many persons who adopt business as a profession are not of the business temperament; and *vice versâ*, there are many persons of business temperament who are not in business, and many of religious temperament who are not in religion. But the person of religious temperament is prone to feel strongly and to express his feelings with even exaggerated emphasis. The emotion may not be deep, nor need it be enduring, but while it lasts it overpowers him and carries him away in a storm of expression. He weeps on small occasion. He groans with remorse. He writhes in contortion of agony at the thought of his sins—or of other people's. Under stress of exhortation and contagion he shrieks and sobs, and may fall down in a faint or even in convulsions. He gushes with pity, especially with self-pity. He sees visions and hears voices. Under stress of emotion he breaks out into eloquence of which his ordinary speech gives little promise. So foreign are these manifestations to his customary behaviour that they may be looked upon as the direct results of inspiration. They are, however, the common features of the religious temperament when it is highly developed.

